# Efavirenz, dysrhythmia and HIV

**DOI:** 10.1002/clc.23745

**Published:** 2021-10-25

**Authors:** Rujittika Mungmunpuntipantip, Viroj Wiwanitkit

**Affiliations:** ^1^ Private Academic Consultant Bangkok Thailand; ^2^ Department of Community Medicine Dr DY Patil University Pune India

To the Editor

We would like to share ideas on “Association between exposure to Efavirenz and substrates of dysrhythmia in HIV‐infected young adults.”^1^ Hosseini et al. concluded, “*patients with Efavirenz had higher prevalence of frequent PVC, frequent PAC, total significant dysrhythmia, Low HRV and prolonged QTc than controls*.”^1^ The Efavirenez is widely mentioned for effect on cardiac rhythm. An important determinant is underlying genetic factors. CYP2B6*6*6 allele carrier is associated with cardiac rhythm problem in person with HIV receiving Efavirenez.^2^ It is interesting to assess background genetic factors in case and control group in the report by Hosseini et al.^1^ In a previous report from Iran, CYP2B6*6 was common in Iran population^3^ and it might be an explanation for the high frequency of cardiac dysrhythmia among HIV cases receiving Efavirenez in the study by Hosseini et al.
